# Does brachytherapy boost improve survival outcomes in Gleason Grade Group 5 patients treated with external beam radiotherapy and androgen deprivation therapy? A systematic review and meta-analysis

**DOI:** 10.1016/j.ctro.2022.10.010

**Published:** 2022-10-29

**Authors:** Terence Tang, Stephanie Gulstene, Eric McArthur, Andrew Warner, Gabriel Boldt, Vikram Velker, David D'Souza, Glenn Bauman, Lucas C. Mendez

**Affiliations:** Department of Radiation Oncology, London Regional Cancer Program, 800 Commissioners Road East, London, Ontario N6A 5W9, Canada

**Keywords:** Prostate cancer, Gleason Grade Group 5, External beam radiotherapy, Brachytherapy boost, Survival outcome

## Abstract

**Background:**

Localized Gleason Grade Group 5 (GG5) prostate cancer has a poor prognosis and is associated with a higher risk of treatment failure, metastases, and death. Treatment intensification with the addition of a brachytherapy (BT) boost to external beam radiation (EBRT) maximizes local control, which may translate into improved survival outcomes.

**Methods:**

A systematic review and meta-analysis was performed to compare survival outcomes for Gleason GG5 patients treated with androgen deprivation therapy (ADT) and either EBRT or EBRT + BT. The MEDLINE (PubMed), EMBASE and Cochrane databases were searched to identify relevant studies. Survival probabilities for distant metastasis-free survival (DMFS), prostate cancer-specific survival (PCSS), and overall survival (OS) were extracted and pooled to create a summary survival curve for each treatment modality, which were then compared at fixed points in time. An additional analysis was performed among studies directly comparing EBRT and EBRT + BT using a random-effects model.

**Results:**

Eight retrospective studies were selected for inclusion, representing a total of 1393 EBRT patients and 877 EBRT + BT patients. EBRT + BT was associated with higher DMFS starting at 6 years (86.8 % vs 78.8 %; p = 0.018) and extending out to 10 years (81.8 % vs 66.1 %; p < 0.001), with an overall hazard ratio of 0.53 (p = 0.02). There was no difference in PCSS or OS between treatment modalities. Differences in toxicity were not assessed. There was a wide range of heterogeneity between studies.

**Conclusion:**

The addition of BT boost is associated with improved long-term DMFS in Gleason GG5 prostate cancer, but its impact on PCSS and OS remains unclear. These results may be confounded by the heterogeneity across study populations with concern for a risk of bias. Therefore, prospective studies are necessary to further elucidate the survival advantage associated with BT boost, which must ultimately be weighed against the toxicity-related implications of this treatment strategy.

## Highlights

• Adding a BT boost to external beam radiation can be used to intensify treatment.
• BT boost improves DMFS but not PCSS or OS in Gleason GG5 prostate cancer.
• There is no prospective data evaluating BT boost in Gleason GG5 disease.

## 1 Introduction

Localized prostate cancer is typically considered an indolent malignancy with a long natural history, with patients often dying of other causes. However, approximately 15 % of patients with localized prostate cancer have high or very high risk disease, which carries an increased risk of recurrence following definitive treatment and subsequent progression to cancer-related death [1], [2].

In particular, Gleason Grade Group 5 (GG5) disease is an independent predictor of a worse prognosis and denotes high or very high risk disease even when other disease characteristics are favorable [3]. Compared to lower Gleason grade groups, GG5 disease has a more dedifferentiated histologic appearance, lacking the morphological characteristics of benign prostate architecture [4]. It is also genetically distinct, harboring genomic instability in pathways that have been linked with androgen deprivation therapy (ADT) resistance [5]. In fact, there is mounting data to suggest that ADT, when combined with definitive radiotherapy, bestows a smaller benefit in Gleason GG5 disease and that Gleason GG5 tumours progress more rapidly to a castrate-resistant state upon recurrence [6], [7].

When treating high risk disease with external beam radiotherapy (EBRT), different methods of treatment intensification are currently available, including the addition of ADT, extending the duration of ADT, and a brachytherapy (BT) boost. BT boost has already been shown to improve biochemical and local disease control in patients with intermediate and high risk disease, though its benefit in the Gleason GG5 subgroup is unclear [8], [9]. However, given recent evidence demonstrating a correlation between local failure and inferior survival [10], [11], BT boost may be particularly important in improving long-term outcomes in Gleason GG5 disease. By maximizing local control within the prostate, BT boost may reduce the risk of future systemic spread and ultimately improve survival outcomes [12]. With this in mind, the present study aims to systematically review the literature to compare survival outcomes in Gleason GG5 patients treated with ADT and either EBRT or EBRT plus BT boost (EBRT + BT).

## 2 Methods

### 2.1 Evidence acquisition

The Preferred Reporting Items for Systematic reviews and meta-Analyses (PRISMA) 2020 statement was used as guideline for this review. A search strategy was first developed using the population, intervention, control, outcome, study design (PICOS) framework (Supplementary Table 1). MEDLINE (PubMed), EMBASE and the Cochrane Central Register of Controlled Trials were then queried to identify relevant articles published between January 1, 2000 and March 29, 2022. The exact search terms utilized are listed in Supplementary Table 2.

**Fig. 1:**
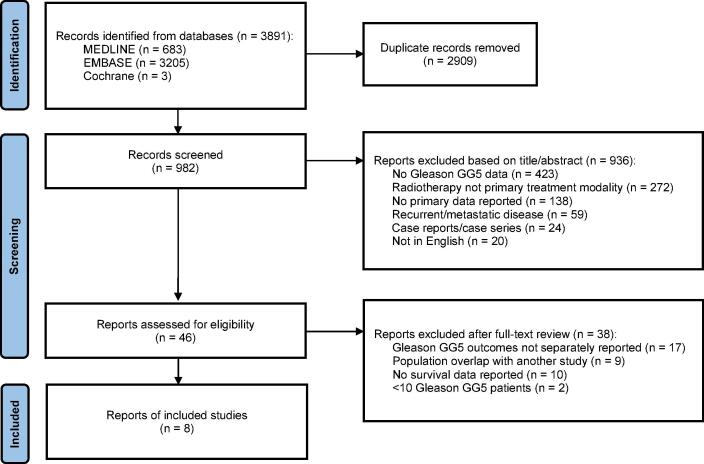
Preferred Reporting Items for Systematic reviews and meta-Analyses (PRISMA) flow diagram. GG5 = Grade Group 5.

After the exclusion of duplicate records, a total of 982 unique results were obtained from MEDLINE (PubMed) and EMBASE and Cochrane. The initial screening of manuscript abstracts, with a brief assessment of full text if necessary, was completed by two authors (SG and TT). Any disagreements were resolved by a third author (LM). Articles were excluded if data for Gleason GG5 disease was not reported; radiotherapy was not the primary treatment modality; patients had recurrent or metastatic disease; or primary data was not reported, as in population database studies. Case reports, case series and articles not in English were also excluded. An in-depth full-text review of 46 manuscripts was then conducted, with additional exclusions made on the basis of: lack of separately reported outcomes for Gleason GG5 disease; overlapping patient cohorts, in which case the broader publication was included; lack of survival data; and total number of Gleason GG5 patients <10. Again, this was initially performed by two authors (SG and TT), with a third author (LM) resolving any discrepancies. The PRISMA flow diagram in Fig. 1 summarizes the search and selection process detailed above.

### 2.2 Data extraction

Data extraction was performed by two authors (SG and TT). Study characteristics were identified, including study type, study design, publication date, enrollment period and sample size. Baseline patient characteristics were also extracted, including median prostate specific antigen (PSA) at diagnosis, clinical *T*-stage at diagnosis, use of pre-treatment staging, median ADT duration, treatment volumes, brachytherapy isotopes and technique, median prostate dose and median number of fractions. In studies that did not separately report baseline characteristics for Gleason GG5 patients, these baseline characteristics were assumed to be similar to those of the overall cohort. Assuming an ⍺/β ratio of 1.5, the equivalent dose in 2-Gy fractions (EQD2Gy) was calculated for the EBRT and BT treatments. The total radiation dose to the prostate for patients receiving EBRT + BT was calculated by summing the EQD2Gy values for the individual EBRT and BT treatments.

Survival probabilities were extracted from the Kaplan-Meier curves for distant metastasis-free survival (DMFS), prostate cancer-specific survival (PCSS) and overall survival (OS) in the included studies, where available. Where mortality functions were reported, the complementary survival probabilities were calculated. In studies reporting survival data amongst specific subgroups, the survival probabilities for each subgroup were extracted. Wherever possible, the number of patients at risk at each time interval were extracted directly from the published studies. In cases where numbers at risk were not reported, these values were estimated using an established method that accounts for censoring [13]. This was performed using the digitized package in R (version 4.0.2) and the DigitizeIt software. In studies directly comparing EBRT to EBRT + BT, a hazard ratio (HR) for the relevant outcomes was also extracted. If HRs were not directly reported in text, they were calculated using the extracted survival estimates and the corresponding numbers at risk. As Yamazaki et al. reported survival data for two separate EBRT groups (i.e., conventional radiotherapy and intensity-modulated radiotherapy), this data was first combined before a HR was calculated with reference to the EBRT + BT group [14].

### 2.3 Risk of bias

The modified Oxford Centre for Evidence-Based Medicine Levels of Evidence criteria were used to assess the quality of each of the included studies [15]. This was performed by one author (SG).

### 2.4 Statistical analysis

Baseline patient and treatment characteristics were summarized for each treatment group using a weighted average of reported medians based on sample size. Survival probabilities were pooled across studies to produce a summary survival curve using a modification of the random-effects meta-analysis method proposed by DerSimonian and Laird [16], [17]. Greenwood’s formula for variance with the delta method was used to estimate the 95 % confidence interval of the summary survival probabilities [18]. Differences between the pooled EBRT and EBRT + BT curves were compared at fixed points in time using log(-log) transformations of the survival functions as associated event times were not available for estimation using the log-rank test [19]. Results were considered statistically significant if p < 0.05. Between-study heterogeneity was calculated for all outcomes by treatment group. Among studies with available HRs, an additional meta-analysis was performed with the RevMan software (version 5.4) using a random-effects model.

## 3 Results

### 3.1 Literature search results

A total of eight studies were selected for inclusion in the meta-analysis, all of which were retrospective series classified as Level 4 evidence [14], [20], [21], [22], [23], [24], [25], [26]. Four reported data from a single institution, while the remaining four were multi-institutional. Including the three studies that reported on both treatment modalities, there were a total of five studies that provided data on outcomes after EBRT and six after EBRT + BT. Among the latter, a low-dose-rate (LDR-BT) boost was used in two studies, a high-dose-rate (HDR-BT) boost in three studies, and either a LDR-BT or HDR-BT in one study.

### 3.2 Patient and treatment characteristics

The baseline patient and treatment characteristics for Gleason GG5 patients from all included studies are summarized in Table 1. There were a total of 1393 patients who received EBRT and 877 patients who received EBRT + BT, with Kishan et al. contributing approximately half of the patients in each group [22]. Median follow-up was 64.5 months in the EBRT group and 71.1 months in the EBRT + BT group. Median age was 69.1 years vs 69.1 years and median pre-treatment PSA was 17.09 ng/mL vs 13.03 ng/mL for the EBRT and EBRT + BT groups, respectively. As for clinical stage, 31 % of EBRT patients were T1-T2a, 31 % T2b-T2c and 38 % T3-T4. Similarly, 33 % of EBRT + BT patients were T1-T2a, 29 % T2b-T2c and 38 % T3-T4. Completion of pre-treatment staging investigations was not specified in any of the EBRT studies but was documented in most of the EBRT + BT studies.

**Table 1:** Baseline patient and treatment characteristics for Gleason GG5 patients in included studies.

	EBRT					EBRT + BT					
Study	Ozyigit et al. [20]	Safdieh et al. [21]	Kishan et al. [22]	Yamazaki et al. [14]	Shilkrut et al. [23]	Kasahara et al. [24]	Tsumura et al. [25]	Tilki et al. [26]	Kishan et al. [22]	Yamazaki et al. [14]	Shilkrut et al. [23]
Patients with Gleason GG5 disease (n)	306	51	734	227	75	18	61	80	436	249	33
Median age (years)	68	73	68	72	73.2	67	69.8	70.3	68	71	66
Median follow-up (months)	70.8	69	61.2	66.3	62.9	53	74	66.1	75.6	66.3	63.6
Median baseline PSA (ng/mL)	29	14.7	9.9	24.3	18.7	26.4	25.94	10.55	9.6	16.13	9.9

*Clinical stage*											
T1-T2a (%)	0	NR	47.5	12.3	49	4.5	0	18.8	48.4	14.9	64
T2b-T2c (%)	55.2	NR	22.2	25.6	28	25.8	0	40.0	30.3	29.7	27
T3-T4 (%)	44.8	NR	30.2	62.1	23	69.7	100	41.3	19.0	55.4	9
Median ADT duration (months)	>24	>24	21.9	9	22	44	48	6	12	42	12
Pelvic radiation (%)	51.6	96	40.7	35.4	96	0	0	NR	73.4	2.4	100
Median EBRT dose (EQD2Gy)	72.32	71.28	74.30	72.00	77.40	50.14	38.57	42.43	NR	38.57	46.80
Median total prostate dose (EQD2Gy)	72.32	71.28	74.30	72.00	77.40	104.14	135.00	91.73	91.50	109.00	101.85

ADT = androgen deprivation therapy; EBRT = external beam radiotherapy; EBRT + BT = external beam radiotherapy plus brachytherapy boost; EQD2Gy = equivalent dose in 2-Gy fractions; GG5 = Grade Group 5; NR = not reported; PSA = prostate specific antigen.

The proportion of patients receiving pelvic nodal irradiation was 47 % and 41 % in the EBRT and EBRT + BT groups, respectively. However, the use of nodal irradiation within the EBRT + BT group may be underestimated, as pelvic lymph node coverage was left to the discretion of the treating physician in Tilki et al. and exact numbers were not reported [26]. ADT was used in all studies and median duration of ADT was 20.3 months vs 23.1 months in the EBRT and EBRT + BT groups, respectively. Median total prostate dose in EQD2Gy was 73.55 Gy for patients receiving EBRT and 100.16 Gy for those receiving EBRT + BT.

### 3.3 Effect of brachytherapy boost on distant metastasis-free survival

**Fig. 2:**
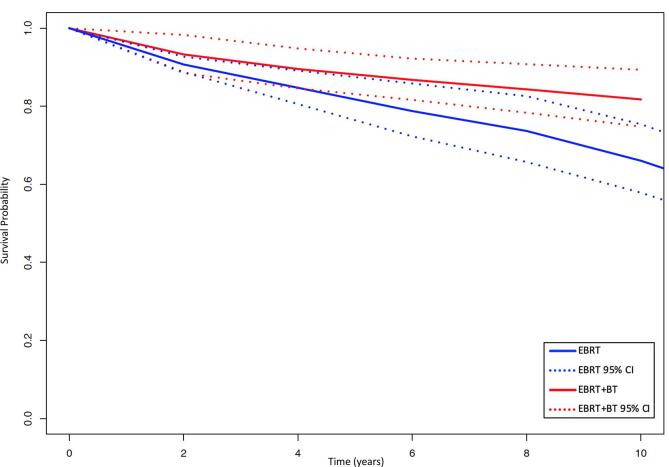
Pooled distant metastasis-free survival curves by treatment group. CI = confidence interval; EBRT = external beam radiotherapy; EBRT + BT = external beam radiotherapy plus brachytherapy boost.

Four studies reported DMFS rates after EBRT and five after EBRT + BT. Fig. 2 displays the pooled survival curves for DMFS among EBRT and EBRT + BT patients. There was an absolute difference in the pooled DMFS estimates favouring EBRT + BT over EBRT at 4 years (89.6 % vs 84.7 %; p = 0.12), with a significant difference at 6 years (86.8 % vs 78.8 %; p = 0.018), 8 years (84.3 % vs 73.7 %; p = 0.010) and 10 years (81.8 % vs 66.1 %; p < 0.001). Between-study heterogeneity was moderate within the EBRT group but low within the EBRT + BT group (Table 2).

**Table 2:** Pooled survival estimates by treatment group.

		DMFS		PCSS		OS	
Treatment		EBRT	EBRT + BT	EBRT	EBRT + BT	EBRT	EBRT + BT
Heterogeneity (%)		40.6	8.0	24.1	50.2	48.8	12.4
4 Years	Pooled Survival Probability (95 % CI)	84.7 (80.6–89.1)	89.6 (84.6–94.8)	94.6 (89.4–100)	96.6 (95.0–98.2)	92.3 (86.8–98.1)	94.5 (92.3–96.9)
	P-Value	0.12		0.37		0.37	

6 Years	Pooled Survival Probability (95 % CI)	78.8 (72.3–85.8)	86.8 (81.6–92.2)	91.5 (84.7–98.9)	93.2 (89.8–96.6)	86.5 (79.3–94.4)	90.0 (86.9–93.3)
	P-Value	0.018		0.62		0.29	

8 Years	Pooled Survival Probability (95 % CI)	73.7 (65.7–82.6)	84.3 (78.4–90.8)	87.0 (79.0–95.9)	88.1 (83.8–92.6)	79.3 (72.1–87.1)	84.9 (74.1–97.4)
	P-Value	0.010		0.78		0.33	

10 Years	Pooled Survival Probability (95 % CI)	66.1 (57.9–75.4)	81.8 (74.8–89.4)	81.4 (72.9–90.8)	82.2 (75.9–89.0)	73.9 (65.7–83.1)	77.4 (61.6–97.3)
	P-Value	<0.001		0.85		0.65	

CI = confidence interval; DMFS = distant metastasis-free survival; EBRT = external beam radiotherapy; EBRT + BT = external beam radiotherapy plus brachytherapy boost; OS = overall survival; PCSS = prostate cancer-specific survival.

**Fig. 3:**
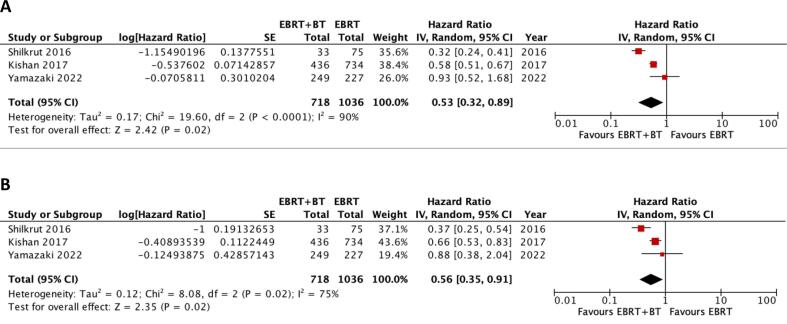
Forest plots for included studies directly comparing EBRT and EBRT + BT. (A) Distant metastasis-free survival. (B) Prostate cancer-specific survival. CI = confidence interval; EBRT = external beam radiotherapy; EBRT + BT = external beam radiotherapy plus brachytherapy boost.

Three studies directly compared DMFS after EBRT and EBRT + BT. The addition of BT boost significantly improved DMFS (HR = 0.53; p = 0.02). Heterogeneity was high with I^2^ = 90 % (Fig. 3A).

### 3.4 Effect of brachytherapy boost on prostate cancer-specific survival

**Fig. 4:**
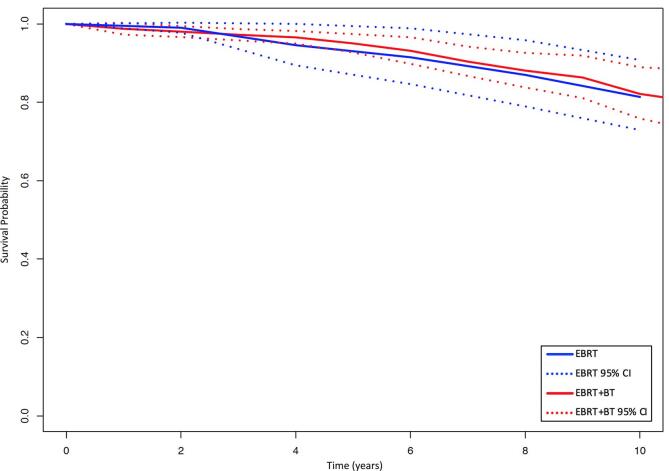
Pooled prostate cancer-specific survival curves by treatment group. CI = confidence interval; EBRT = external beam radiotherapy; EBRT + BT = external beam radiotherapy plus brachytherapy boost.

Four studies reported PCSS rates after EBRT and five after EBRT + BT. Fig. 4 displays the pooled survival curves for PCSS among EBRT and EBRT + BT patients. There was an absolute difference in the pooled PCSS estimates favouring EBRT + BT over EBRT at 4 years (96.6 % vs 94.6 %; p = 0.37), 6 years (93.2 % vs 91.5 %; p = 0.62), 8 years (88.1 % vs 87.0 %; p = 0.78) and 10 years (82.2 % vs 81.4 %; p = 0.85) without ever reaching statistical significance. Between-study heterogeneity was low within the EBRT group but moderate within the EBRT + BT group (Table 2).

Three studies directly compared PCSS after EBRT and EBRT + BT. The addition of BT boost significantly improved PCSS (HR = 0.56; p = 0.02). Heterogeneity was high with I^2^ = 75 % (Fig. 3B).

### 3.5 Effect of brachytherapy boost on overall survival

**Fig. 5:**
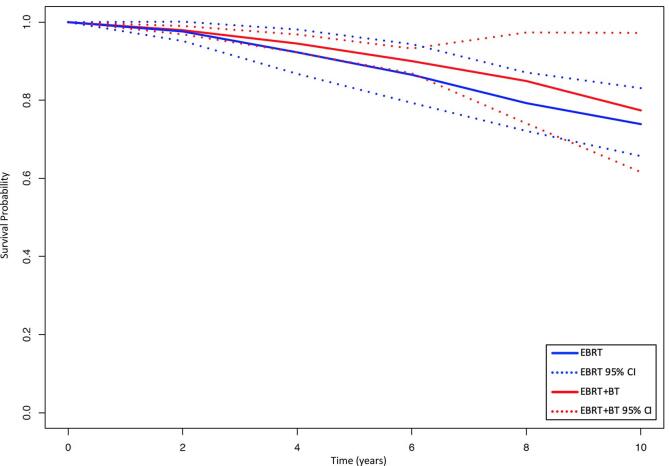
Pooled overall survival curves by treatment group. CI = confidence interval; EBRT = external beam radiotherapy; EBRT + BT = external beam radiotherapy plus brachytherapy boost.

Four studies reported OS rates after EBRT and five after EBRT + BT. Fig. 5 displays the pooled survival curves for OS amongst EBRT and EBRT + BT patients. There was a trend towards improved OS with EBRT + BT at 4 years (94.5 % vs 92.3 %; p = 0.37), 6 years (90.0 % vs 86.5 %; p = 0.29), 8 years (84.9 % vs 79.3 %; p = 0.33) and 10 years (77.4 % vs 73.9 %; p = 0.65). Between-study heterogeneity was moderate within the EBRT group and low within the EBRT + BT group (Table 2).

Two studies directly compared OS after EBRT and EBRT + BT. There was a trend toward improved OS with BT boost (HR = 0.86; p = 0.08), but this was heavily weighted towards Kishan et al. with only a minor contribution from Yamazaki et al. (not shown) [14], [22].

## 4 Discussion

This meta-analysis of eight retrospective studies suggested a DMFS benefit with the addition of BT boost in the treatment of Gleason GG5 disease. This was true in both the pooled survival curve and pooled HR analyses. Differences in DMFS were first observed at six years post-treatment and extended out to ten years. This finding is consistent with other studies evaluating the effect of BT boost in Gleason GG5 prostate cancer. In a multi-institutional retrospective series of approximately 1000 patients, Foster et al. identified an increase in freedom from distant metastases with the addition of BT boost, which was most pronounced in the subset of Gleason GG5 patients with additional poor-prognosis risk factors [27]. A National Comprehensive Cancer Network (NCCN) analysis of 467 patients also found that BT boost improved freedom from metastasis at five years (89 % vs 67 %; p < 0.05) [28].

In localized prostate cancer, DMFS has been shown to be a surrogate endpoint for overall survival [29]. However, while there was a trend toward improved OS with EBRT + BT in this meta-analysis, no significant differences were identified. In addition, despite a significant benefit in PCSS with EBRT + BT in the pooled HR analysis, this was not replicated in the pooled survival curve analysis possibly owing to differences in sample size and heterogeneity. A recent Surveillance, Epidemiology, and End Results (SEER) database study similarly did not identify significant differences in either prostate cancer-specific mortality or overall mortality between EBRT and EBRT + BT on multivariate analysis [30]. Likewise, ASCENDE-RT and other randomized trials have failed to demonstrate an overall survival advantage with the addition of BT boost in high risk prostate cancer, though these studies were not powered to show differences in this endpoint [8], [9], [31], [32], [33]. Ultimately, while BT boost appears to improve DMFS, its impact on PCSS and OS in Gleason GG5 patients remains unclear.

As far as the rationale behind how BT boost could reduce the rate of metastasis, it has been hypothesized that by minimizing the risk of local failure, BT boost can limit the risk of a subsequent wave of systemic spread [11], [12]. Findings from a meta-analysis of six randomized trials by Kishan et al. support this concept, as local failure following definitive radiotherapy was associated with worse DMFS as well as PCSS and OS [10]. This was corroborated by another meta-analysis of 18 randomized trials by Ma et al. wherein local failure after definitive radiotherapy was found to be an independent prognosticator of DMFS, PCSS and OS in high risk patients [11]. Some recent work has also shown that BT boost appears to stimulate a different profile of immune response when compared to EBRT [34], [35], although it is unclear if this difference in immunomodulation has a role in reducing metastatic events.

The current American Society of Clinical Oncology (ASCO)/Cancer Care Ontario (CCO) guidelines support the use of BT boost in high risk prostate cancer [36]. However, among this heterogeneous group of patients, it is unclear who may benefit most. This meta-analysis suggests Gleason GG5 disease is a strong indication for BT boost, though the relevance of the other NCCN high risk factors is unclear. Most patients in this meta-analysis had T1-T2 disease with a median pre-treatment PSA under 25, similar to patients from the randomized trials upon which the ASCO/CCO guidelines are based [9], [37], [38]. As for higher *T*-stages, there is retrospective evidence to suggest that BT boost is still important in T3b-T4 disease [39], although this population is underrepresented in randomized trials. BT boost also appears to improve outcomes in the subset of high risk patients with additional poor prognostic factors [27], [40], though a recent cohort study of patients with very high risk disease has called that into question [41]. Ultimately, in the absence of randomized data, all patients with high risk disease should still be considered for BT boost provided there are no other contraindications.

As for the relevance of age, the overall median age of patients in this meta-analysis was approximately 70, suggesting that patients in this age range still stand to benefit from BT boost. A recent retrospective study demonstrated patients above the age of 70 treated with BT boost did not have worse oncologic outcomes compared to their younger counterparts [42]. A subgroup analysis comparing patients 71–80 to those over 80 also identified similar oncologic outcomes. The only exception was inferior OS in those over 80, likely driven by higher rates of all-cause mortality associated with increasing age [42]. Thus, when considering BT boost in elderly patients, particularly in octo- and nonagenarians [43], overall life expectancy should be assessed using the Charlson comorbidity score or a similar scale [44] and weighed against the long-term benefits of this treatment strategy. Outstanding medical comorbidities that may limit elderly patients’ ability to physically undergo BT boost must also be considered.

It is also important to contextualize the potential benefits of BT boost with the risk of additional treatment-related toxicity. In the periprocedural period, there is a risk of BT-associated complications like urinary retention requiring prolonged catheterization, which can have a short-term impact on patients’ quality of life. The risk of such complications, particularly urinary retention, is often related to specific baseline patient characteristics [45], which should be carefully assessed and discussed at the time of initial consultation to identify the best candidates for BT boost. In terms of longer-term toxicities, the ASCENDE-RT trial did find that the addition of a LDR-BT boost resulted in increased genitourinary (GU) and gastrointestinal (GI) toxicity events [46]. Fortunately, many of these side effects were temporary, but the cumulative incidence of grade 3 GU toxicity was still significantly higher in the BT boost arm at five years post-treatment (18.4 % vs 5.2 %; p < 0.01), with urethral stricture constituting approximately half of these events [46]. This may be particularly relevant for GG5 patients as higher Gleason score was a predictive factor for significant late GU toxicity, in addition to age and severity of pre-treatment urinary symptoms [46]. In contrast to the increased morbidity demonstrated in ASCENDE-RT, another randomized trial in a similar population of patients did not identify any increased Grade 3 + GU toxicity when using a HDR-BT boost up to 8 years post-treatment (13 % vs 7 %; p = 0.2) [33]. In addition, a study directly comparing HDR-BT versus LDR-BT boost found lower toxicity rates with the former, though this was a small and non-randomized study [47]. A larger, single institutional retrospective review also showed a trend towards lower rates of toxicity with HDR-BT versus LDR-BT boost, though this did not reach statistical significance [48]. In this context, a systematic review concluded that HDR-BT boost was well-tolerated and serious complications were rare, with weighted aggregate estimates of 2.1 % and 0.2 % for late grade 3 + GU and GI toxicity, respectively [49]. Despite this, attempts to minimize toxicity should always be considered, including optimizing procedural technique, limiting dose to organs-at-risk and offering effective counselling and interventions [49]. Overall, current data suggests that additional toxicity with BT boost is low, especially when using HDR-BT, and supports a favorable therapeutic ratio for the addition of BT boost in appropriately-selected high risk patients.

As the first meta-analysis of its kind to date, this study provides further support for the use of BT boost in the radiation treatment of Gleason GG5 disease. These findings are applicable to the modern era of radiotherapy as all included studies were published after the year 2015 and utilized long-term ADT. However, this meta-analysis does have several limitations. All included studies were retrospective in nature, as there were no prospective series or randomized trials reporting separate oncological outcomes for Gleason GG5 disease in this context. Retrospective studies have inherent biases, including selection bias, which may confound the observed differences between EBRT + BT and EBRT. Additionally, Kishan et al. alone contributed more than half of the patients in both the EBRT and EBRT + BT groups [22]. This was unavoidable given the limited number of studies on this topic. Finally, there was a fair degree of heterogeneity amongst the included studies, and while a random-effects model was used, this limits the robustness of the pooled estimates and may very well account for the lack of observed differences in PCSS and OS between treatment modalities.

## 5 Conclusion

In conclusion, this meta-analysis provides evidence that the addition of BT boost to EBRT and ADT improves DMFS in Gleason GG5 prostate cancer, but its effect on PCSS and OS remains unclear. However, these results may be confounded by the heterogeneous study populations and the overall risk of bias across studies. As this meta-analysis did not compare toxicity outcomes between treatment modalities, it remains unclear if BT boost results in additional toxicity. Ultimately, given the lack of high-level evidence available, prospective trials are needed to further evaluate the survival benefits of this treatment strategy and contextualize them against the toxicity-related implications of BT boost.

## Funding

None.

## Declaration of Competing Interest

The authors declare that they have no known competing financial interests or personal relationships that could have appeared to influence the work reported in this paper.
