# Supplementary material for: Does brachytherapy boost improve survival outcomes in Gleason Grade Group 5 patients treated with external beam radiotherapy and androgen deprivation therapy? A systematic review and meta-analysis
Source: Clin Transl Radiat Oncol. 2022 Oct 29;38:21–7. doi: 10.1016/j.ctro.2022.10.010 (PMC9637706; doi:10.1016/j.ctro.2022.10.010)
Supplement: Supplementary data 1 [file mmc1.docx]

**Supplementary Table 1:** PICOS search strategy.

| Population | Localized Gleason GG5 prostate cancer |
| --- | --- |
| Intervention | External beam radiotherapy with brachytherapy boost |
| Control | External beam radiotherapy |
| Outcome | Metastasis-free survival, prostate cancer-specific survival, overall survival |
| Study Design | Prospective or retrospective series |
