# Supplementary material for: Does brachytherapy boost improve survival outcomes in Gleason Grade Group 5 patients treated with external beam radiotherapy and androgen deprivation therapy? A systematic review and meta-analysis
Source: Clin Transl Radiat Oncol. 2022 Oct 29;38:21–7. doi: 10.1016/j.ctro.2022.10.010 (PMC9637706; doi:10.1016/j.ctro.2022.10.010)
Supplement: Supplementary data 2 [file mmc2.docx]

**Supplementary Table 2:** Search terms.

| MEDLINE (PubMed) | (gleason[tw] OR poorly differentiated[tw] OR high grade[tw]) AND (proton therapy[mh] OR external beam radiotherapy[tw] OR ebrt[tw] OR external radiation[tw] OR external radiotherapy[tw]) AND (prostate[tw] OR prostatic neoplasms[mh] OR prostatic[tw])  Limits: 2000-present |
| --- | --- |
| EMBASE and Cochrane (via OVID) | (exp Gleason score/ or gleason.mp. or poorly differentiated.mp. or high grade.mp.)  and  (proton therapy.mp. or exp proton therapy/ or external beam radiotherapy.mp. or exp external beam radiotherapy/ or ebrt.mp. or external radiation.mp. or external radiotherapy.mp.)  and  (exp prostate cancer/ or exp prostate tumor/ or prostate.mp. or exp prostate/ or prostatic.mp.)  Limits: Exclude Medline Records.  2000-present |
